# Prognostic Value of FDG PET/CT in Patients with Nodal Peripheral T-Cell Lymphoma

**DOI:** 10.3390/diagnostics13172834

**Published:** 2023-09-01

**Authors:** Woo Hee Choi, Eun Ji Han, Joo Hyun O, Eun Kyoung Choi, Joon-Il Choi, Gyeongsin Park, Byung-Ock Choi, Young-Woo Jeon, Gi-June Min, Seok-Goo Cho

**Affiliations:** 1 Division of Nuclear Medicine, Department of Radiology, College of Medicine, The Catholic University of Korea, Seoul 06591, Republic of Korea; wh0522@catholic.ac.kr (W.H.C.); ojoohyun@songeui.ac.kr (J.H.O.); eet0224@gmail.com (E.K.C.); 2 Department of Radiology, College of Medicine, The Catholic University of Korea, Seoul 06591, Republic of Korea; dumkycji@gmail.com; 3 Department of Hospital Pathology, College of Medicine, The Catholic University of Korea, Seoul 06591, Republic of Korea; gspark@catholic.ac.kr; 4 Department of Radiation Oncology, College of Medicine, The Catholic University of Korea, Seoul 06591, Republic of Korea; choibo67@catholic.ac.kr; 5 Department of Hematology, College of Medicine, The Catholic University of Korea, Seoul 06591, Republic of Korea; native47@catholic.ac.kr (Y.-W.J.); beichest@catholic.ac.kr (G.-J.M.); chosg@catholic.ac.kr (S.-G.C.)

**Keywords:** peripheral T-cell lymphoma, FDG PET/CT, Deauville score, prognosis

## Abstract

This study evaluated the prognostic significance of FDG PET/CT in patients with nodal peripheral T-cell lymphoma (PTCL). We retrospectively reviewed patients with histologically confirmed nodal PTCL who underwent FDG PET/CT at baseline, after three cycles of first-line chemotherapy (interim), and at the end of therapy. Response was assessed visually using the Deauville 5-point scale (D5PS); scores of 1, 2, and 3 were considered PET-negative, and scores of 4 and 5 were considered PET-positive. The associations between FDG PET/CT findings and survival were assessed using Cox regression analysis. A total of 79 patients (44 males and 35 females; median age 56 years) were included in this study. In response assessment, 17 (22%) had an interim PET-positive result and 10 (13%) had an end-of-therapy PET-positive result. During a median follow-up of 50 months, 37 patients (47%) presented with disease progression and 30 patients (38%) died. The estimated 5-year progression-free survival (PFS) and overall survival (OS) were 57% and 64%, respectively. An interim PET-positive result was the only significant indicator of PFS. Higher International Prognostic Index and end-of-therapy PET-positive result were significant independent prognostic factors of OS. Interim and end-of-therapy FDG PET/CT responses based on D5PS are meaningful in predicting the outcomes of patients with nodal PTCL.

## 1. Introduction

Peripheral T-cell lymphoma (PTCL) is an aggressive lymphoid neoplasm originating from mature or post-thymic T cells and accounts for 5–10% of non-Hodgkin’s lymphoma (NHL) cases in Western countries and 15–20% of NHL in Asian countries. PTCL is a clinically, morphologically, and genetically heterogeneous group of diseases with complex pathobiology. Primary nodal PTCL is the most frequent subtype and includes PTCL not otherwise specified (PTCL-NOS), angioimmunoblastic T-cell lymphoma (AITL), and anaplastic lymphoma kinase positive (ALK+) and negative (ALK−) anaplastic large-cell lymphoma (ALCL) [1,2,3]. While cyclophosphamide, hydroxydaunomycin, vincristine, and prednisone (CHOP) and CHOP-like regimens are commonly used as a first-line chemotherapy, patients with PTCL, except those with ALK+ ALCL, generally have poor response to conventional chemotherapy and aggressive clinical course. Current guidelines recommend up-front autologous stem cell transplantation for most patients because salvage chemotherapy is often ineffective [4,5].

The International Prognostic Index (IPI) is a widely used tool for risk stratification of patients with aggressive NHL [6]. Age > 60 years, Eastern Cooperative Oncology Group (ECOG) performance status ≥ 2, Ann Arbor stage III or IV, elevated serum lactate dehydrogenase (LDH), and involvement of more than one extranodal site are poor prognostic factors. Although this traditional IPI helps to predict the prognosis of some subtypes of PTCL, it has unsatisfactory performance in some other subtypes, such as PTCL-NOS and AITL [1,7]. Several modified IPI models have been proposed for effective prognostication of PTCL, but independent validation on a large series is warranted [8].

F-18-fluoro-2-deoxyglucose (FDG) positron emission tomography (PET)/computed tomography (CT) is considered essential for assessing initial staging and treatment response in most aggressive types of lymphoma because of its superior diagnostic accuracy compared with CT. Most of the guidelines recommend FDG PET/CT at baseline and at the end of therapy. Interim FDG PET/CT after a few cycles of chemotherapy is a promising tool for the early prediction of outcome and personalized care in Hodgkin’s lymphoma (HL). However, interim FDG PET/CT is carefully recommended in diffuse large B-cell lymphoma (DLBCL) because of the possibility of false-positive results [9,10,11]. Most PTCL cases are FDG-avid, and FDG PET/CT is recommended over conventional CT due to its high diagnostic accuracy in clinical practice [5,12]. However, the prognostic significance of FDG PET/CT has not been fully explored in patients with PTCL. Since PTCL has a poor prognosis and extreme heterogeneity, it is necessary to identify more effective prognostic markers than IPI. Therefore, we evaluated the prognostic value of FDG PET/CT in patients with nodal PTCL.

## 2. Materials and Methods

### 2.1. Patients

Patients with histologically confirmed nodal PTCL who underwent FDG PET/CT at baseline (b-PET), after three cycles of first-line chemotherapy (interim PET, i-PET), and after completion of chemotherapy (end-of-therapy PET, e-PET) between 2011 and 2019 were retrospectively reviewed. This study was approved by the institutional review board of Catholic Medical Center (IRB no. XC21RIDI0017), and the need for patient consent for this retrospective review of imaging studies and clinical data was waived. This study was performed in accordance with the relevant guidelines and regulations of the ethical committee.

### 2.2. FDG PET/CT Acquisition

All patients fasted for at least 4 h. FDG (222–555 MBq) was injected intravenously, and scanning began approximately 60 min later. No subjects had a blood glucose level above 200 mg/dL before the injection. No intravenous contrast agent was administered. Studies were acquired on integrated PET/CT scanners, Biograph Truepoint (Siemens Medical Solutions, Knoxville, TN, USA) and Discovery 710 (GE Healthcare, Milwaukee, WI, USA). All patients were in a supine position. CT began at the vertex or orbitomeatal line and progressed to the upper thigh using a standard protocol of 120 kV, 50 mA, 5 mm slice thickness (Biograph Truepoint), and 120 kVp, with variable mAs adjusted by topographic image and 2.5 mm slice thickness (Discovery 710). PET followed immediately over the same body region. Acquisition time was 1.5–2.5 min per bed position. The CT data were used for attenuation correction, and PET images were reconstructed using standard-ordered-subset expectation maximization.

### 2.3. Image Analysis

All FDG PET/CT images were initially reviewed by two experienced nuclear medicine physicians who were blinded to the patients’ survival information. We visually assessed the bone marrow (BM) status on b-PET as positive or negative. BM PET-positive was defined as discrete FDG uptake, either single or multiple, in the bone(s) or heterogeneously increased uptake with intensity visually greater than that of the patient’s liver. BM PET-negative was defined as no or diffuse uptake in the bone(s) without discrete uptake [13]. Response was assessed using the Deauville 5-point scale (D5PS). Deauville score 1 indicates no uptake, score 2 indicates uptake less than or equal to that of the mediastinum, score 3 indicates uptake greater than the mediastinum but less than the liver, score 4 indicates uptake moderately greater than the liver, and score 5 indicates uptake markedly greater than the liver or any new lesion [14]. Scores 1, 2, and 3 were defined as PET-negative, and scores 4 and 5 were considered PET-positive. If there was disagreement on visual assessment between two readers, consensus was achieved with a third nuclear medicine physician [11].

### 2.4. Statistical Analysis

Agreement between diagnostic methods was tested using Cohen κ values. A ĸ value of 0.0–0.2 was considered to represent poor agreement; 0.21–0.4, fair; 0.41–0.6, moderate; 0.61–0.8, substantial; and 0.81–1.0, almost perfect. Progression-free survival (PFS) was defined as the time from the date of b-PET/CT imaging for diagnosis to disease progression/relapse, death, or final follow-up. Overall survival (OS) was defined as the time from the date of b-PET/CT imaging to death or final follow-up. The Kaplan–Meier method was used to estimate the survival times, and the log-rank test was used for assessment of differences in survival between groups. From the results of the univariate analysis, multivariate Cox regression analysis was performed to identify the independent prognostic factors of PFS and OS. All statistical analyses were performed using SPSS version 26.0 (IBM Corp., Armonk, NY, USA) and MedCalc Statistical Software version 20.023 (MedCalc Software bvba, Ostend, Belgium). Differences were considered statistically significant when the *p* value was less than 0.05.

## 3. Results

### 3.1. Patient Characteristics

A total of 87 patients with nodal PTCL treated with full cycle of first-line chemotherapy combined with three FDG PET/CT studies were initially identified; 8 patients were excluded due to absence of the target lesion following excision (*n* = 3) or low FDG avidity (*n* = 5) in b-PET. The remaining 79 patients (44 males, 35 females; median age 56 years) were included in this study. At baseline, the majority (*n* = 64, 81%) had advanced-stage disease, and BM involvement was confirmed through biopsy of the posterior iliac crest in 25 patients (32%). One patient failed to undergo BM biopsy (BMB). The general characteristics of patients are presented in Table 1.

### 3.2. Assessment of FDG PET/CT

In b-PET images of the 79 patients, 12 (15%) had BM PET-positive results with discrete focal FDG uptake in the bone(s), and only 2 of these had bony abnormalities on enhanced CT at baseline. The remaining 67 (85%) had BM PET-negative results: 48 (61%) showed BM with FDG uptake with an intensity similar to or less than the patient’s liver and 19 (24%) showed diffusely increased FDG uptake in the BM with an intensity greater than the liver. Concordance analysis showed poor agreement between visual assessment of BM FDG uptake and BMB (Cohen’s ĸ = −0.105, *p* = 0.288), with 32 discordant cases (40%) (Table 2). Using BMB as the gold standard for BM involvement, the estimated sensitivity, specificity, positive predictive value, and negative predictive value of b-PET were 8%, 83%, 18%, and 66%, respectively.

**Figure 1:**
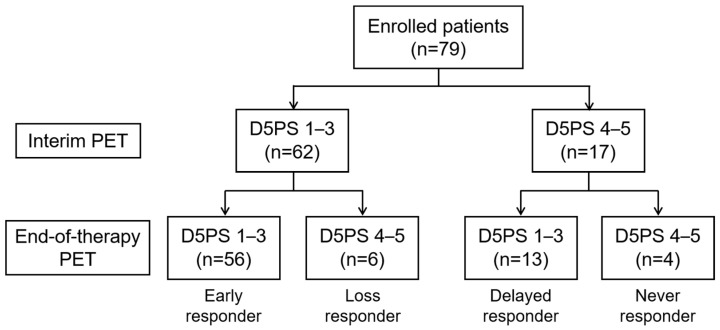
Response assessment at i-PET and e-PET.

In the response assessment of the 79 patients, i-PET was negative in 62 (78.5%), and e-PET was negative in 69 (87%). Of the 17 patients with positive i-PET, 4 (24%) also had positive e-PET (Figure 1, Table S1). Of the 62 patients with negative i-PET, e-PET-positive results were observed in 6 patients (10%).

### 3.3. Survival Analysis and Prognostic Value

During a median follow-up of 50 months (range 6–115), 37 patients (47%) exhibited disease progression, and 30 patients (38%) died. The estimated 5-year PFS and OS were 57% and 64%, respectively. Of the 37 patients with disease progression, 6 were diagnosed with disease progression on e-PET images. All eight eligible patients with ALK+ ALCL remained alive without disease progression over a median follow-up of 58 months. Of the 79 patients, 26 (33%) who achieved first remission underwent autologous hematopoietic stem cell transplantation (HSCT); 11 (14%) underwent allogeneic HSCT in refractory or relapsed disease; and 42 (53%) were treated with chemotherapy only. No statistically significant differences were observed in OS between these three groups (mean OS time 58, 80, and 61 months, respectively; *p* > 0.05).

**Figure 2:**
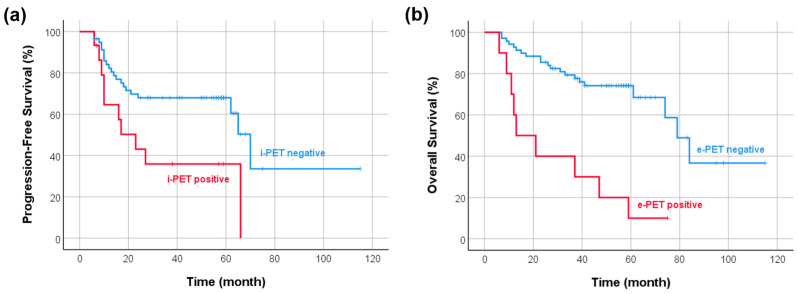
Kaplan–Meier curves of PFS according to metabolic response on i-PET and OS according to metabolic response on e-PET. (**a**) Patients with positive i-PET had worse PFS than patients with negative i-PET (median PFS 23 vs. 70 months, *p* = 0.014). (**b**) Patients with positive e-PET showed poorer OS than patients with negative e-PET (median OS 13 vs. 79 months, *p* < 0.001).

**Figure 3:**
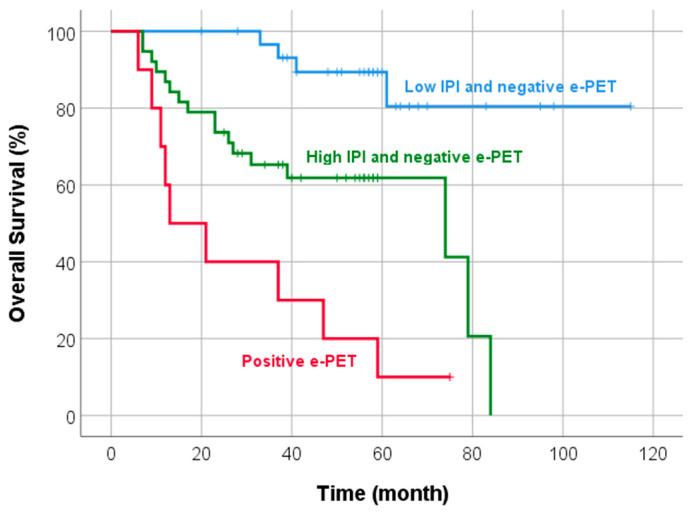
Kaplan–Meier curve of OS according to IPI score combining e-PET response. Patients with a low IPI score and negative e-PET result had better OS than patients with a high IPI score and negative e-PET or with positive e-PET (mean OS 101 vs. 56 vs. 29 months, *p* < 0.05).

**Figure 4:**
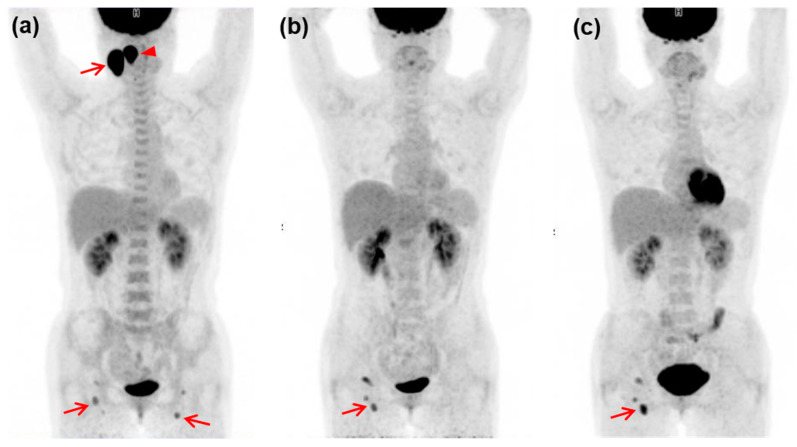
A 43-year-old female patient with PTCL-NOS, with IPI score of 1. B-PET image (**a**) shows intensely increased FDG uptake in the right palatine tonsil (SUVmax 12.8, arrowhead) and right cervical level II lymph node (SUVmax 17.6, arrow). In addition, lesser intense FDG uptake was noted in right cervical level I–II and bilateral inguinal nodes (arrows). Both i-PET (**b**) and e-PET (**c**) images were positive (Deauville score of 4), with persistently increased FDG uptake in the right inguinal nodes (arrows). The patient had a PFS of two months and an OS of nine months.

In univariate analysis, histologic subtypes other than ALK+ ALCL and positive i-PET were associated with inferior PFS, while higher IPI score, positive BM uptake in b-PET, and positive e-PET were associated with inferior OS (Table 3). In multivariate analysis, positive i-PET was the only significant indicator in predicting PFS (hazard ratio (HR) 2.688, 95% confidence interval (CI) 1.254–5.761). Higher IPI score (HR 3.182, 95% CI 1.425–7.106) and positive e-PET (HR 5.567, 95% CI 2.497–12.410) were independent prognostic factors of OS (Figure 2). We further divided patients into three prognostic subgroups based on IPI score and e-PET result: group 1 with a low IPI score and negative e-PET result (*n* = 31); group 2 with a high IPI score and negative e-PET result (*n* = 38); and group 3 with positive e-PET result (*n* = 10). Group 2 had worse OS than group 1 (mean OS 56 vs. 101 months, *p* = 0.001). Group 3 showed poorer OS than group 1 (mean OS 29 vs. 101 months, *p* < 0.001) and group 2 (median OS 13 vs. 74 months, *p* = 0.006) (Figure 3 and Figure 4).

## 4. Discussion

In this study, we evaluated the prognostic role of FDG PET/CT at baseline and during and after completion of first-line chemotherapy in patients with nodal PTCL. Our results demonstrated that i-PET response based on D5PS had significant value in predicting PFS, and e-PET response had significant value in predicting OS.

With independent prognostic value, interim FDG PET/CT response-adapted therapy is now a standard of care for patients with HL [15]. However, the prognostic role of interim FDG PET/CT in NHL remains controversial. Even in DLBCL, the negative predictive value of interim FDG PET/CT for survival is consistently high, but the positive predictive value is variable [16]. Several studies on the prognostic value of interim and end-of-therapy FDG PET/CT in PTCL have been performed; however, these studies were retrospective in design and had a small or heterogeneous population. In previous studies of PTCL patients with various histologic subtypes, a positive interim FDG PET/CT result predicted inferior PFS and OS [17,18]. Another study of 45 patients with only AITL showed that interim FDG PET/CT had a significant prognostic value for predicting PFS and OS [19]. Conversely, El-Galay et al. [20] reported that interim FDG PET/CT was not predictive of outcome in 124 patients with PTCL. In our results, negative i-PET result was significantly associated with superior PFS but not OS. Further studies with a prospective design and a larger sample size are required to better clarify these conflicting results for the prognostic value of interim FDG PET/CT.

The D5PS is an internationally recommended scale for PET/CT response assessment of FDG-avid lymphoma. A Deauville score of 3 is considered to indicate a good response to treatment in HL and most B-cell lymphomas, such as DLBCL and FL [21]. However, the optimal cut-off score predicting prognosis in T-cell lymphoma is controversial. Moon et al. suggested that it was appropriate to regard a score of 3 as positive in the interim FDG PET/CT assessment of AITL patients [19]. El-Galay et al. reported that the interim response, which classified score >3 as positive, was not correlated with PFS and OS in patients with PTCL [20]. Changing the cut-off for positivity to score 5 showed a strong tendency toward worse prognosis. In our study, the score 3 group showed a favorable outcome compared with the score 4 or 5 groups, in both interim and end-of-therapy response. Further studies will be needed to better clarify the best D5PS for positivity in PTCL.

In our study, higher IPI score and positive e-PET results were significant independent prognostic factors of OS. The IPI is a powerful prognostic tool for predicting the outcome of aggressive NHL. PTCL represents a heterogeneous group of aggressive lymphomas and is generally associated with poor prognosis compared to B-cell lymphomas. CHOP or CHOP-like chemotherapy has been considered to be the frontline standards; however, it has unsatisfactory results in terms of response rate and survival. For this reason, more effective new treatment strategies are being developed, and more accurate prognostic prediction is important [22]. Previous studies reported that IPI is a good prognostic predictor in PTCL, but other studies have questioned the prognostic value of IPI in PTCL and several modified IPI models have been proposed for the effective prognostication of PTCL [8]. The IPI is based only on clinical characteristics at baseline, and the response to treatment is not included. FDG PET/CT is now considered the most accurate tool for assessing remission in aggressive NHL [9,10,11]. In subgroup analysis of our study, combining IPI score with e-PET added prognostic value. Patients with a low IPI score and negative e-PET result had better OS than patients with a high IPI score and negative e-PET or positive e-PET (mean OS 101 vs. 56 vs. 29 months, *p* < 0.05). Combining clinical IPI scores and FDG PET/CT metabolic response to treatment could improve prognostic prediction and could enhance the likelihood of appropriate management in patients with PTCL.

BM involvement is one of the most important prognostic factors in patients with lymphoma [23]. Previous studies reported that baseline PET/CT is more sensitive than BMB for the detection of BM involvement in DLBCL and HL [24,25]. However, El-Galay et al. reported low sensitivity (18%) of FDG PET/CT in detecting BM infiltration in patients with PTCL [20], consistent with our results (8%). Low-volume (10–20%) diffuse BM involvement on histology was reported to cause false-negative PET findings [26]. Several studies have suggested that FDG PET/CT could not replace BMB in T-cell lymphoma [20,27,28,29]. In our results, a BM PET-positive result was associated with inferior OS, although this association was not observed in multivariate analysis. Both BMB and FDG PET/CT are complementary in the evaluation of bone involvement in patients with PTCL. Heterogeneously increased or intense discrete BM uptake(s) is considered as BM involvement in FDG PET/CT of lymphoma. However, the interpretation of diffusely increased BM uptake is controversial [27,29,30]. We considered our 19 patients with diffusely increased BM uptake higher than that of the liver as BM PET-negative. In subgroup analysis, patients with diffusely increased BM uptake showed significantly better OS than patients with focal BM uptake (*p* = 0.037), but no significant difference was observed between patients with diffusely increased BM uptake and patients with no BM uptake (*p* = 0.920). Similarly, Chen et al. reported the focal BM uptake pattern is a better prognostic factor than BMB in DBLCL [31]. Further studies will be needed on the selection of an appropriate cut-off for BM involvement of lymphoma in FDG PET/CT. 

The main limitation of this study was its retrospective design. Our study included FDG PET/CT images obtained at two institutions with different PET/CT scanners. We adopted visual analysis rather than quantitative measures to reduce the variability across scanners and imaging sites. In addition, all FDG PET/CT images were centrally reviewed without knowledge of the scanner, BMB result, and patient outcome. Other limitations included the small sample size and heterogeneity of histological subtypes. PTCL is a relatively rare and heterogeneous group, and most PTCL studies have these limitations. In addition, our inclusion criteria were relatively strict to ensure paired interim and end-of-therapy comparison. The heterogeneity of PTCL subtypes may limit the prognostic performance of FDG PET/CT. In addition, this study included patients who received two different regimens (ProMACE-cytaBOM and CHOP/CHOP-like) as first-line chemotherapy. There was no significant difference in IPI score between the two treatment groups, and the type of regimen did not affect the prognosis in survival analysis.

## 5. Conclusions

Interim FDG PET/CT response was a significant prognostic factor of PFS in patients with nodal PTCL. End-of-therapy FDG PET/CT response was a significant prognostic factor of OS, regardless of IPI. Combining IPI and end-of-therapy FDG PET results improved the stratification of PTCL patients. Therefore, interim and end-of-therapy FDG PET/CT could facilitate the accurate prediction of clinical outcomes and enhance the likelihood of appropriate management in patients with nodal PTCL.

## Figures and Tables

**Table 1:** Characteristics of the 79 patients.

Variables		No. of Patients (%)
Age	Median (range)	56 years (18–77)
Sex	Male	44 (56%)
	Female	35 (44%)
ECOG PS	0–1	70 (89%)
	2–4	9 (11%)
Histologic type	PTCL-NOS	34 (43%)
	AITL	29 (37%)
	ALK+ ALCL	8 (10%)
	ALK− ALCL	8 (10%)
BMB result ^1^	Negative	53 (67%)
	Positive	25 (32%)
Extranodal involvement	0–1	41 (52%)
	>1	38 (48%)
Ann Arbor stage	I	2 (3%)
	II	13 (16%)
	III	18 (23%)
	IV	46 (58%)
LDH level	Normal	29 (37%)
	Elevated	50 (63%)
IPI score	0–2	37 (47%)
	3–5	42 (53%)
Regimen of first-line chemotherapy	ProMACE-cytaBOM	35 (44%)
	CHOP or CHOP-like	44 (56%)

ECOG PS: Eastern Cooperative Oncology Group performance status; PTCL-NOS: peripheral T-cell lymphoma-not otherwise specified; AITL: angioimmunoblastic T-cell lymphoma; ALK: anaplastic lymphoma kinase; ALCL: anaplastic large cell lymphoma; BMB: bone marrow biopsy; LDH: lactic acid dehydrogenase; IPI: International Prognostic Index; proMACE-cytaBOM: prednisone, cyclophosphamide, adriamycin, and etoposide and cytarabine, bleomycin, vincristine, and methotrexate; CHOP: cyclophosphamide, adriamycin, vincristine, and prednisone. ^1^ Not available in one patient.

**Table 2:** Visual BM assessment on baseline PET and BMB results.

	BMB-Negative	BMB-Positive	Total
**BM PET-negative**	44 (56%)	23 (29%)	67 (85%)
**BM PET-positive**	9 (11%)	2 (3%)	11 (14%)
**Total**	53 (67%)	25 (32%)	78 (99%) ^1^

BMB: bone marrow biopsy; BM: bone marrow; PET: positron emission tomography. ^1^ BMB result was not available in one patient (1%).

**Table 3:** Univariate analysis for PFS and OS.

Variables	PFS (*n* = 73) ^1^		OS (*n* = 79)	
	HR (95% CI)	*p*	HR (95% CI)	*p*
Age (≤60 vs. >60 y)	0.8304 (0.3878–1.7778)	0.632	1.2977 (0.5958–2.8267)	0.512
Sex (male vs. female)	1.1823 (0.5736–2.4368)	0.650	1.3043 (0.6285–2.7069)	0.476
Histologic subtype (ALK+ ALCL vs. others)	–	0.024 *	–	0.056
ECOG PS (0–1 vs. 2–4)	0.7608 (0.2106–2.7487)	0.677	2.9094 (0.6867–12.3267)	0.147
Stage (I, II vs. III, IV)	1.2740 (0.5203–3.1196)	0.596	2.1161 (0.8931–5.0140)	0.089
LDH (normal vs. elevated)	1.0158 (0.4816–2.1426)	0.967	1.9541 (0.9399–4.0626)	0.073
BMB (negative vs. positive)	0.8450 (0.3811–1.8735)	0.678	1.2712 (0.5700–2.8349)	0.558
Extranodal involvement (0–1 vs. >1)	1.0105 (0.4879–2.0925)	0.978	1.9534 (0.9438–4.0428)	0.071
IPI score (0–2 vs. 3–5)	1.2913 (0.6227–2.6777)	0.492	2.7874 (1.3412–5.7929)	0.006 *
Regimen of chemotherapy (proMACE-cytaBOM vs. CHOP/CHOP-like)	0.6433 (0.3128–1.3228)	0.230	1.3352 (0.6469–2.7561)	0.434
BM uptake in b-PET (negative vs. positive)	2.3968 (0.7622–7.5369)	0.135	4.8748 (1.4339–16.5730)	0.011 *
i-PET (negative vs. positive)	3.3864 (1.2833–8.9359)	0.014 *	1.5582 (0.6303–3.8520)	0.337
e-PET (negative vs. positive)	1.1937 (0.2417–5.8948)	0.828	19.7452 (5.2663–74.0324)	<0.001 *

PFS: progression-free survival; OS: overall survival; HR: hazard ratio; ALK: anaplastic lymphoma kinase; ALCL: anaplastic large cell lymphoma; ECOG PS: Eastern Cooperative Oncology Group performance status; LDH: lactic acid dehydrogenase; BMB: bone marrow biopsy; IPI: International Prognostic Index; proMACE-cytaBOM: prednisone, cyclophosphamide, adriamycin, and etoposide and cytarabine, bleomycin, vincristine, and methotrexate; CHOP: cyclophosphamide, adriamycin, vincristine, and prednisone; BM: bone marrow; PET: positron emission tomography. ^1^ Six patients whose disease progression was confirmed in e-PET were excluded from analysis of PFS due to suspected progressive disease after the fifth cycle of chemotherapy. * *p* < 0.05.

## Data Availability

The data from this study are available from the corresponding author upon reasonable request.

## Supplementary Materials

The following supporting information can be downloaded at: https://www.mdpi.com/article/10.3390/diagnostics13172834/s1, Table S1: Number of patients in each Deauville score category at i-PET and e-PET.
