# Supplementary material for: Prognostic Value of FDG PET/CT in Patients with Nodal Peripheral T-Cell Lymphoma
Source: Diagnostics (Basel). 2023 Sep 1;13(17):2834. doi: 10.3390/diagnostics13172834 (PMC10487142; doi:10.3390/diagnostics13172834)
Supplement: Supplementary file 1 [file diagnostics-13-02834-s001.zip › diagnostics-2569031-supplementary.pdf]

Table S1. Number of patients in each Deauville score category at i-PET and e-PET.

		End-of-therapy PET					
		Score 1	Score 2	Score 3	Score 4	Score 5	Total
**Interim PET**	Score 1	28	0	0	0	1	29 (37%)
	Score 2	12	10	0	1	1	24 (30%)
	Score 3	1	3	2	2	1	9 (11%)
	Score 4	3	3	5	2	1	14 (18%)
	Score 5	1	1	0	0	1	3 (4%)
	Total	45 (57%)	17 (22%)	7 (9%)	5 (6%)	5 (6%)	79 (100%)
